# Supplementary material for: Cytoplasmic Inter-Subunit Interface Controls Use-Dependence of Thermal Activation of TRPV3 Channel
Source: Int J Mol Sci. 2019 Aug 16;20(16):3990. doi: 10.3390/ijms20163990 (PMC6719031; doi:10.3390/ijms20163990)
Supplement: Supplementary file 1 [file ijms-20-03990-s001.pdf]

Supplementary Information

Cytoplasmic intersubunit interface controls use-dependence of thermal activation of TRPV3 channel

Lucie Macikova, [1,2] Lenka Vyklicka, [1] Ivan Barvik, [3] Alexander I. Sobolevsky, [4] Viktorie Vlachova [1,*]

[1] Department of Cellular Neurophysiology, Institute of Physiology Czech Academy of Sciences, Prague, Czech Republic

[2] Department of Physiology, Faculty of Science, Charles University, Prague, Czech Republic

[3] Division of Biomolecular Physics, Institute of Physics, Faculty of Mathematics and Physics, Charles University, Prague, Czech Republic

[4] Department of Biochemistry and Molecular Biophysics, Columbia University, New York, NY, USA

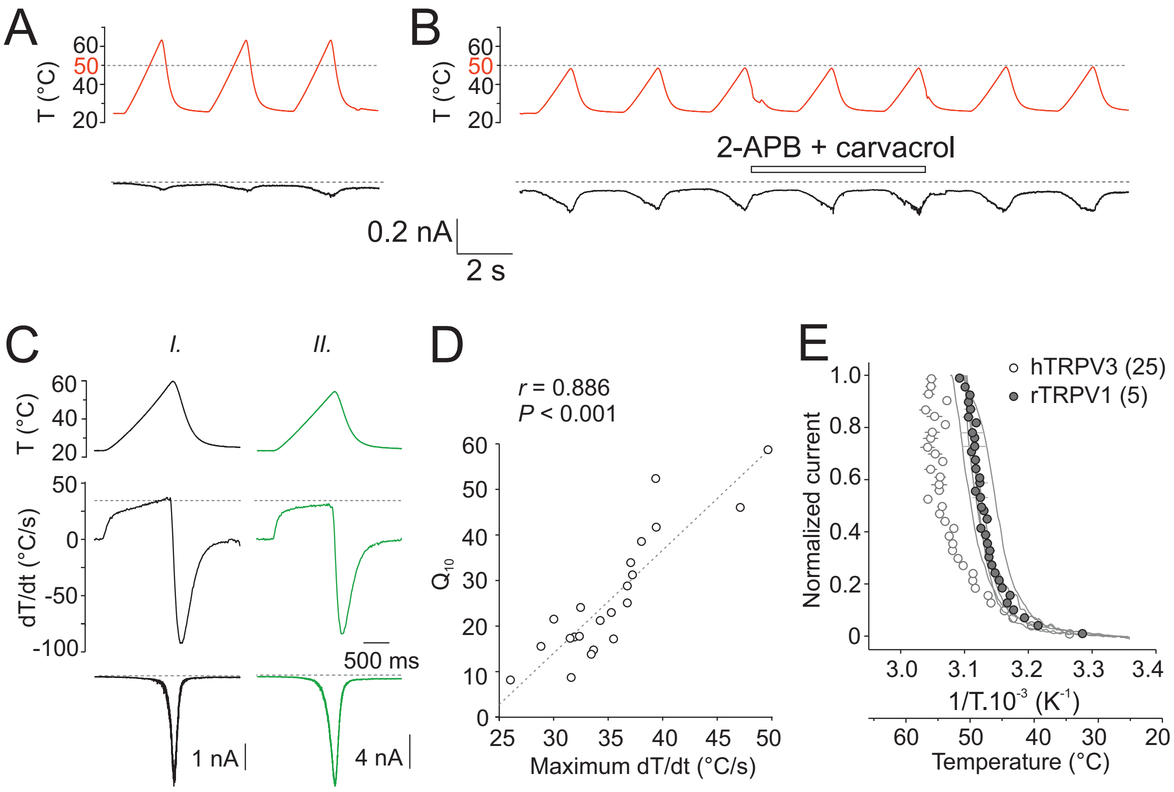

Figure S1. The activation threshold and the slope sensitivity of TRPV3 temperature dependence vary with the speed of temperature exchange. (A) Untransfected HEK293T cells do not exhibit any specific sensitivity to heat (>60 °C) or the combination of 2-APB and carvacrol (B). (C) Shown are typical time courses of the 1st derivative (middle traces) of heat stimuli (upper traces) with resulting heat-induced currents (lower traces) for two different cells (I and II). (D) The time course of heat stimulus affects the Q10 values. The Q10 values linearly correlate with the respective maximum of the 1st derivative of the heat stimuli (Pearson product-moment correlation coefficient, $r = 0.886$; $n = 22$). (E) Heat-induced currents measured from wild-type rat TRPV1, normalized to maximum. The average currents are shown as grey circles with grey bars indicating S.E.M.. The average currents from human TRPV3 are shown for comparison (solid circles with grey bars indicating S.E.M.; the number of cells is indicated in parentheses).

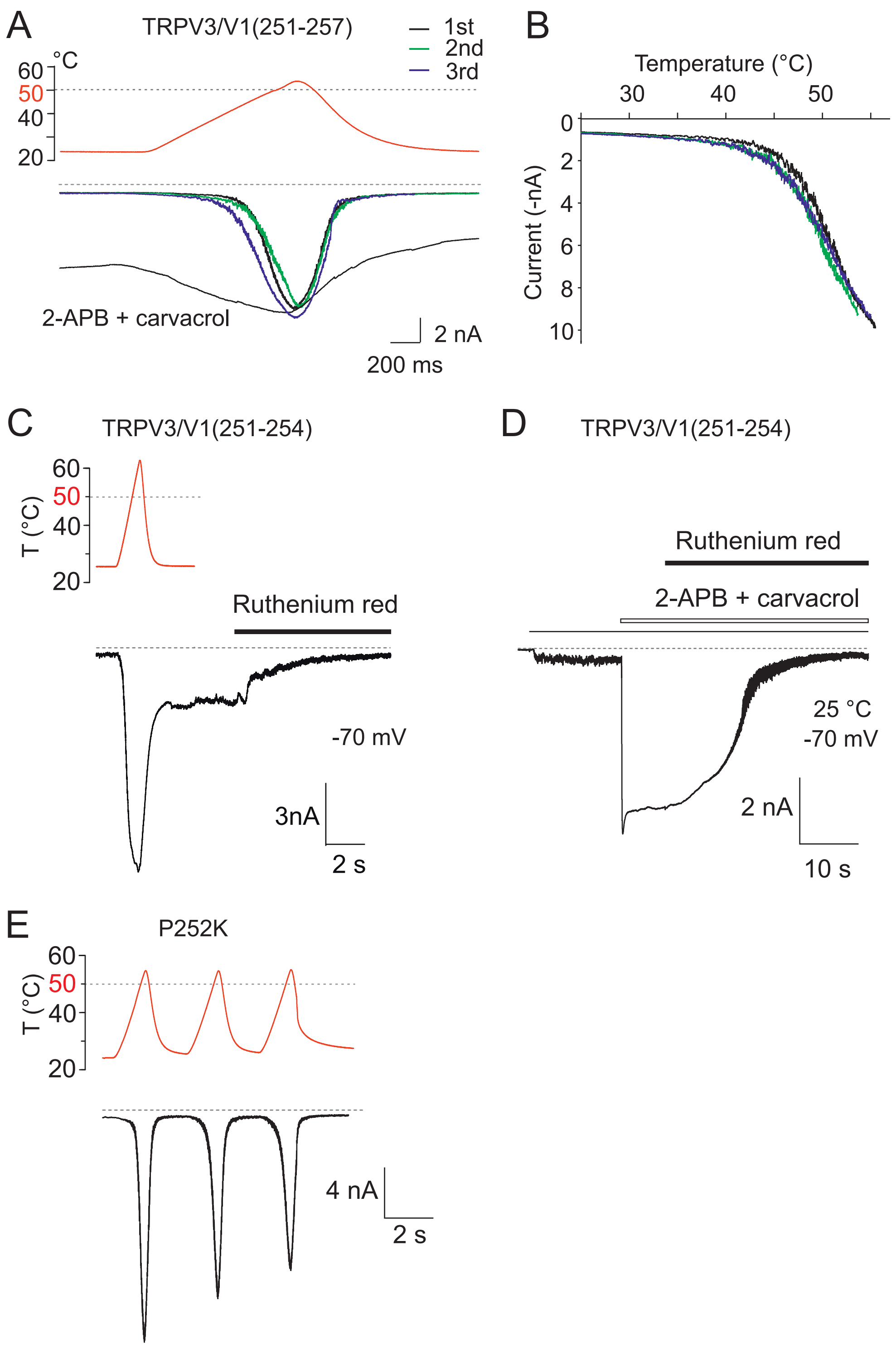

Figure S2. Mutations within the ARD-TRPV3 finger 3 induce sensitized phenotypes. (A) Expanded traces of the first four heat responses (the fourth recorded in the presence of agonists, 100 μM 2-APB + 100 μM carvacrol) for the recording shown in Fig. 2A. (B) Current-temperature relationship of the initial three heat responses shown in A. (C) Representative whole-cell current recorded at -70 mV from chimeric TRPV3/V1(251-254) channels evoked by heat stimulus (shown above). The current persisted after cessation of the heat stimulus and was fully blocked by ruthenium red (10 μM). (D) Immediately after whole-cell formation, extracellular solution containing divalent cations (160 mM NaCl, 2.5 mM KCl, 1 mM CaCl2, 2 mM MgCl2, 10 mM HEPES, 10 mM glucose) was switched to divalent cation free solution (150 mM NaCl, 5 mM EGTA, 10 mM HEPES, horizontal line above the record). The cells were stimulated with mixed agonists (100 μM 2-APB + 100 μM carvacrol). Ruthenium red (10 μM) blocked the currents in the presence of agonists. Holding potential, -70 mV. (E) Mutation at proline 252 is primarily responsible for the sensitized phenotype of the chimeric triple mutant of TRPV3/V1(251-254). Note that the P252K mutant desensitizes upon repeated heat stimulation.

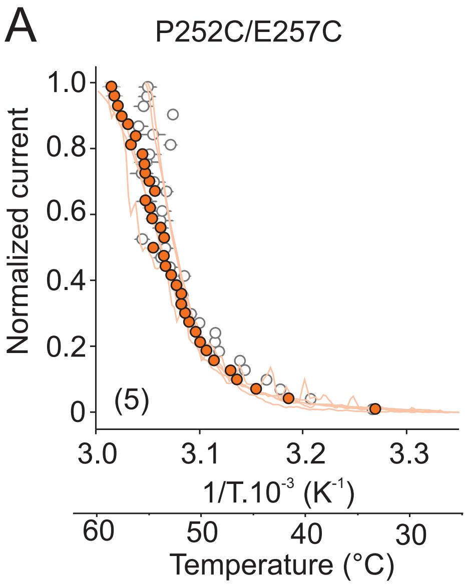

Figure S3. Summary of mutagenesis experiments in which the finger 3 loop was modified. (A-D) Heat responses from HEK293T cells expressing the indicated mutants, normalized to the maximum amplitude with average values overlaid (colored circles with gray bi-directional error bars indicating S.E.M.). The average current for wild-type TRPV3 is overlaid for comparison as empty circles with grey bars indicating mean ± S.E.M. Number of cells is given in parentheses.

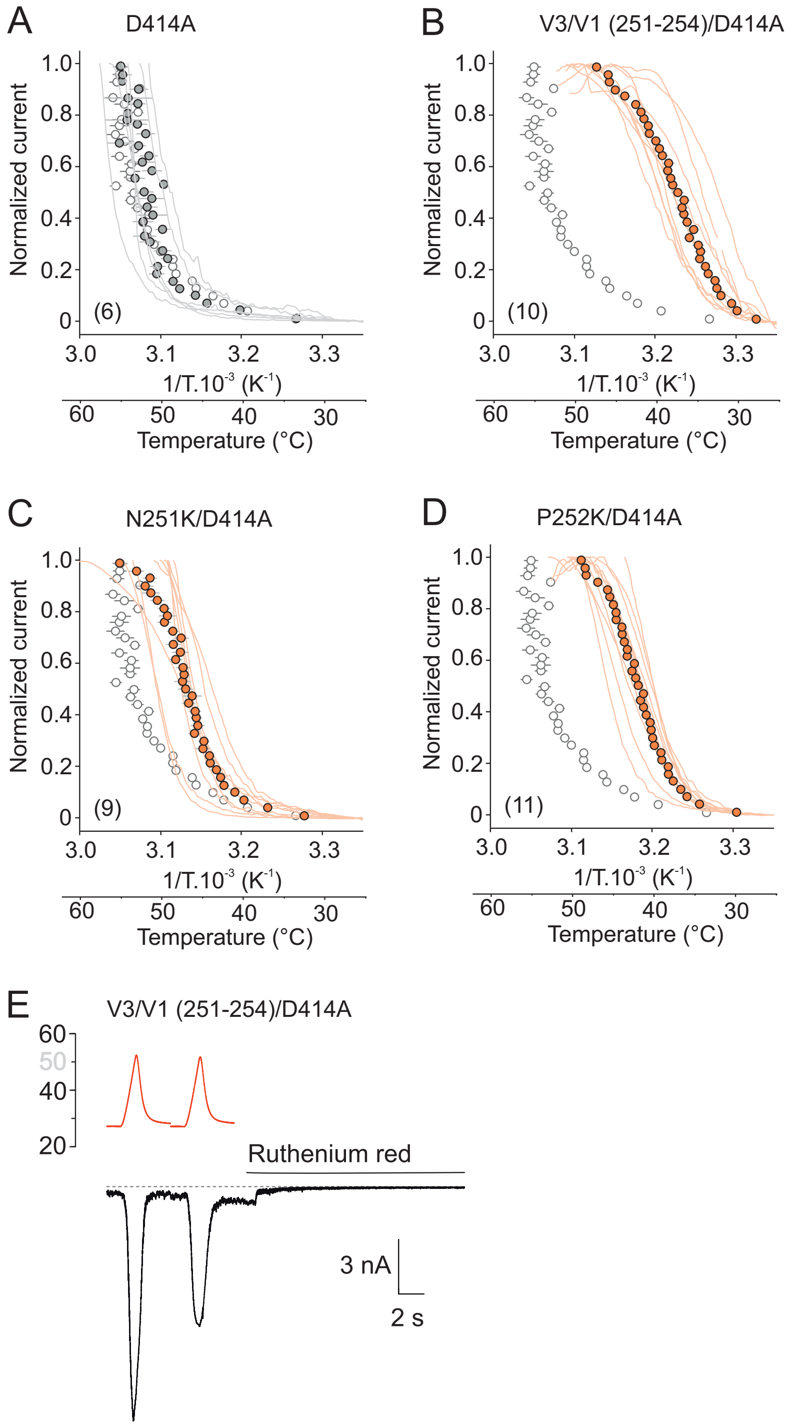

Figure S4. Substituted residues in finger 3 do not directly interact with the MPD. (A-D) Heat responses from HEK293T cells expressing the indicated mutants, normalized to the maximum amplitude with average values overlaid (colored circles with gray bi-directional error bars indicating ± S.E.M.). The average current for wild-type TRPV3 is overlaid for comparison as empty circles with grey bars indicating mean ± S.E.M. Number of cells is given in parentheses. (E) Representative whole-cell recording from the combined mutant TRPV3/V1(251-254)/D414A measured at -70 mV. The construct phenocopied the sensitized properties of the parent template.

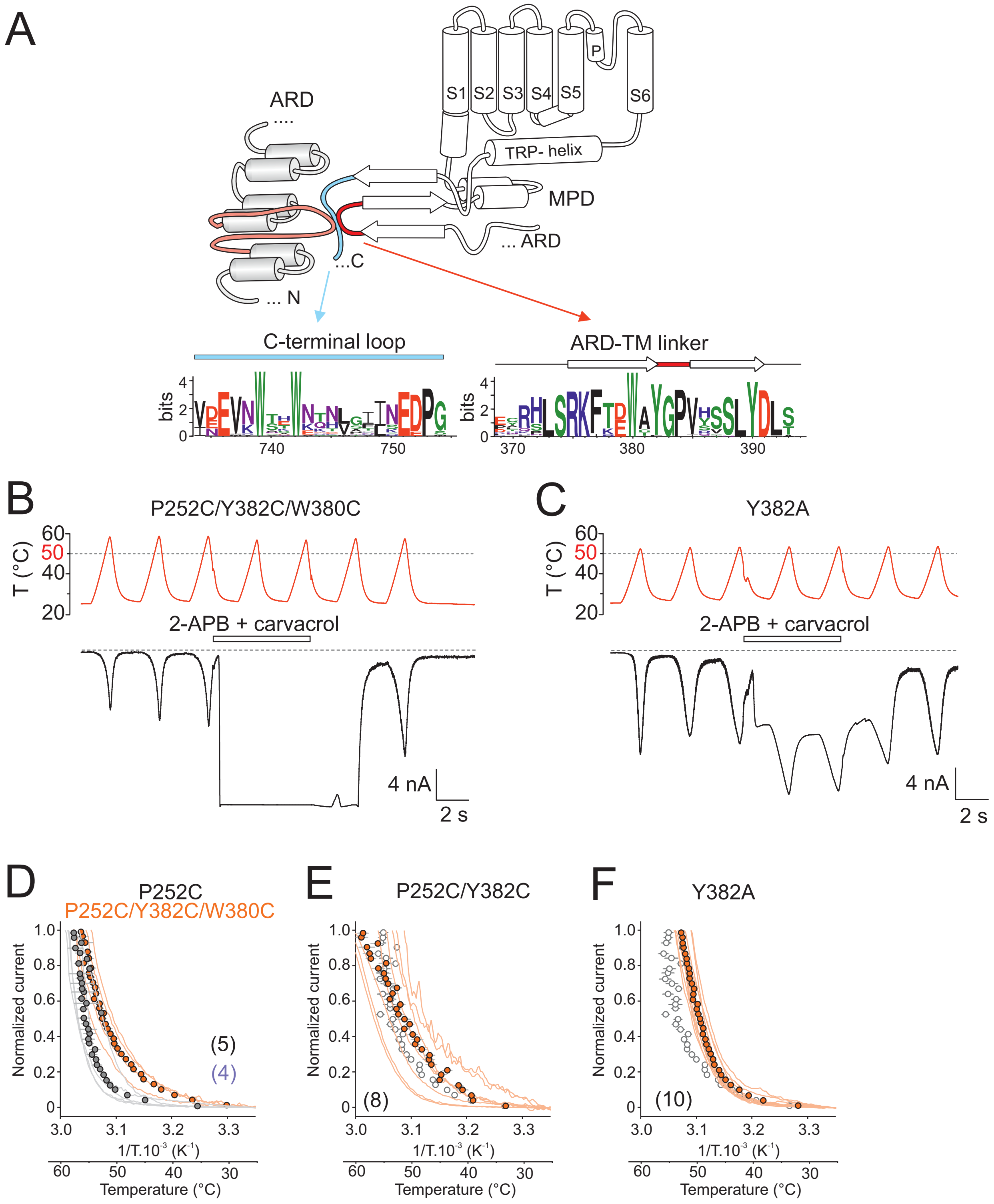

Figure S5. Mutations at the interface between ARD and MPD. (A) Topology diagram of TRPV3 N-terminal finger 3 (salmon) positioned in close proximity of the loop from the ankyrin repeat domain-transmembrane domain (ARD-TM) linker region (red) and the C-terminal loop domain (light blue) of an adjacent subunit. MPD, membrane proximal domain. Below, amino acid conservation of these regions indicating the positions of highly conserved W380 and Y382 in the ARD-TM linker. (B and C) Representative whole-cell recording from the combined (B) triple cysteine mutant P252C/Y382C/W380C and (C) the single mutant Y382A, both measured at -70 mV. (D-F) Heat responses from HEK293T cells expressing the indicated mutants, normalized to the maximum amplitude with average values overlaid (colored circles with gray bi-directional error bars indicating S.E.M.). The average current for wild-type TRPV3 is overlaid for comparison as empty circles with grey bars indicating mean ± S.E.M. Number of cells is given in parentheses.

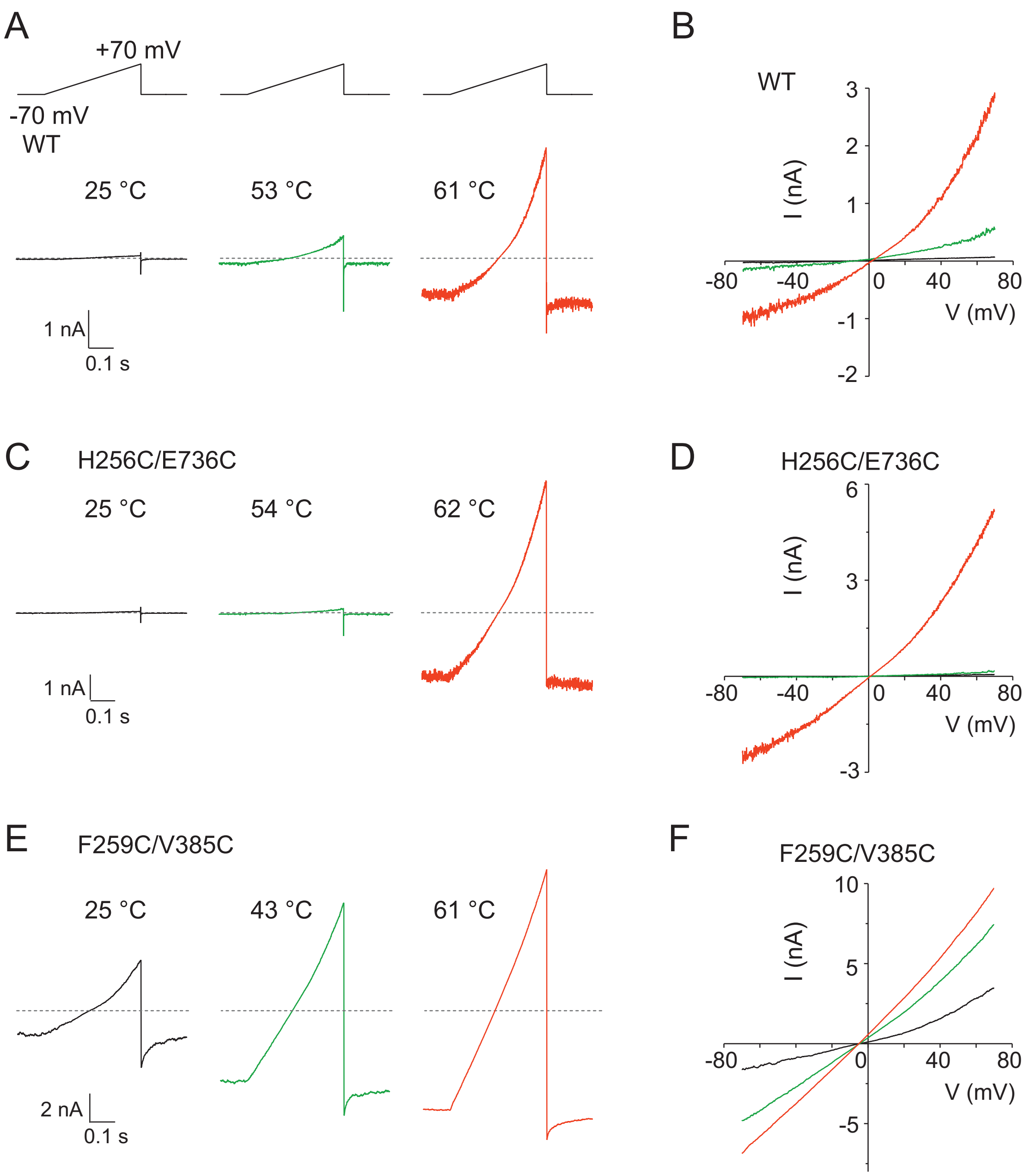

Figure S6. Activation of double cysteine mutants by voltage and temperature. (A) Representative voltage ramp (−70 to +70 mV, 400 ms) traces recorded in control extracellular solution from HEK293T cells expressing wild-type TRPV3 at 25 °C (black), 43-53°C (green) and 61-62°C (red). The voltage ramps were applied immediately after the temperature was settled at the indicated temperature. (B) Current-to-voltage (I/V) relationship from recording shown in (A). Representative voltage ramp traces and I/V relationships from a cell expressing H256C/E736C (C and D) or F259C/V385C (E and F), measured at indicated temperatures. These experiments were performed in triplicate with similar results for each construct.

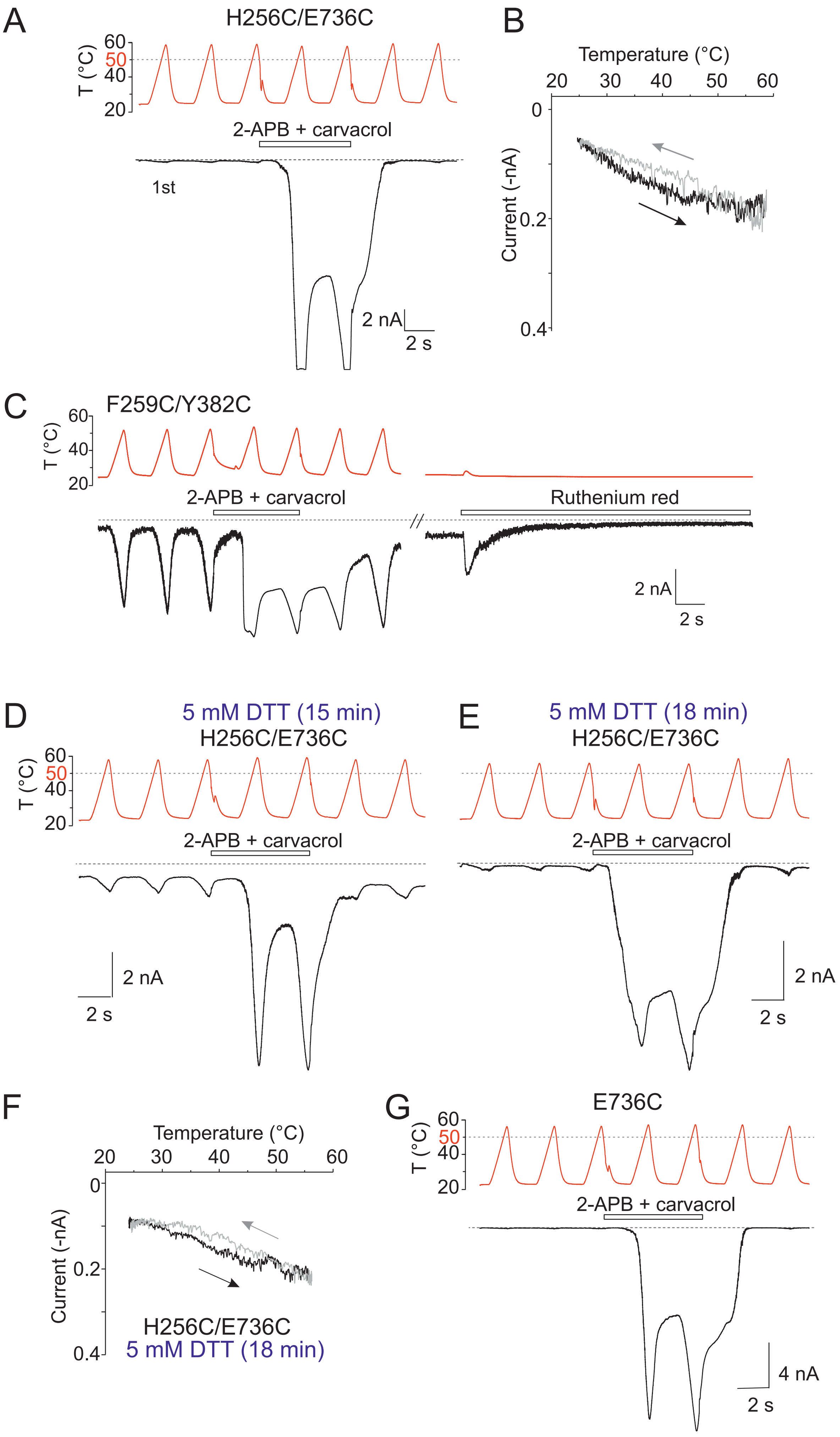
A
H256C/E736C
T (°C)
60
50
40
20
2-APB + carvacrol
1st
2 nA
2 s
B
Temperature (°C)
20
30
40
50
60
Current (-nA)
0
0.2
0.4
C
F259C/Y382C
T (°C)
60
40
20
2-APB + carvacrol
Ruthenium red
2 nA
2 s
D
5 mM DTT (15 min)
H256C/E736C
T (°C)
60
50
40
20
2-APB + carvacrol
2 nA
2 s
E
5 mM DTT (18 min)
H256C/E736C
2-APB + carvacrol
2 nA
2 s
F
Temperature (°C)
20
30
40
50
60
Current (-nA)
0
0.2
0.4
H256C/E736C
5 mM DTT (18 min)
G
E736C
T (°C)
60
50
40
20
2-APB + carvacrol
4 nA
2 s

Figure S7. Recordings to disulfide locking experiments. (A) Representative whole-cell currents evoked by repetitive heat stimuli (shown above the records) recorded from HEK293T cell expressing the H256C/E736C double cysteine mutant channels. Currents were recorded in the absence or presence of mixed agonists (100 μM 2-APB + 100 μM carvacrol), as indicated by the horizontal bar above the records. Holding potential, -70 mV. Notice that responses in the presence of agonists exceeded the lower input range limit of the amplifier. (B) Current-temperature relationship for the first heat response shown in A. (C) Representative whole-cell currents recorded at -70 mV, evoked by heat stimuli (shown above) in the absence or presence of mixed agonists (100 μM 2-APB + 100 μM carvacrol), recorded from the indicated double cysteine mutant of TRPV3. The channels exhibited basal currents that were fully blocked by ruthenium red (10 μM). (D and E) Whole-cell currents recorded at -70 mV evoked by heat, recorded from the H256C/E736C-expressing cells pretreated with dithiothreitol (DTT) for 15-18 min. (F) Current-temperature relationship of the first heat response shown in E. (G) The control single cysteine mutation E736C produces heat-resistant phenotype (compare with A above and Fig. 6A).

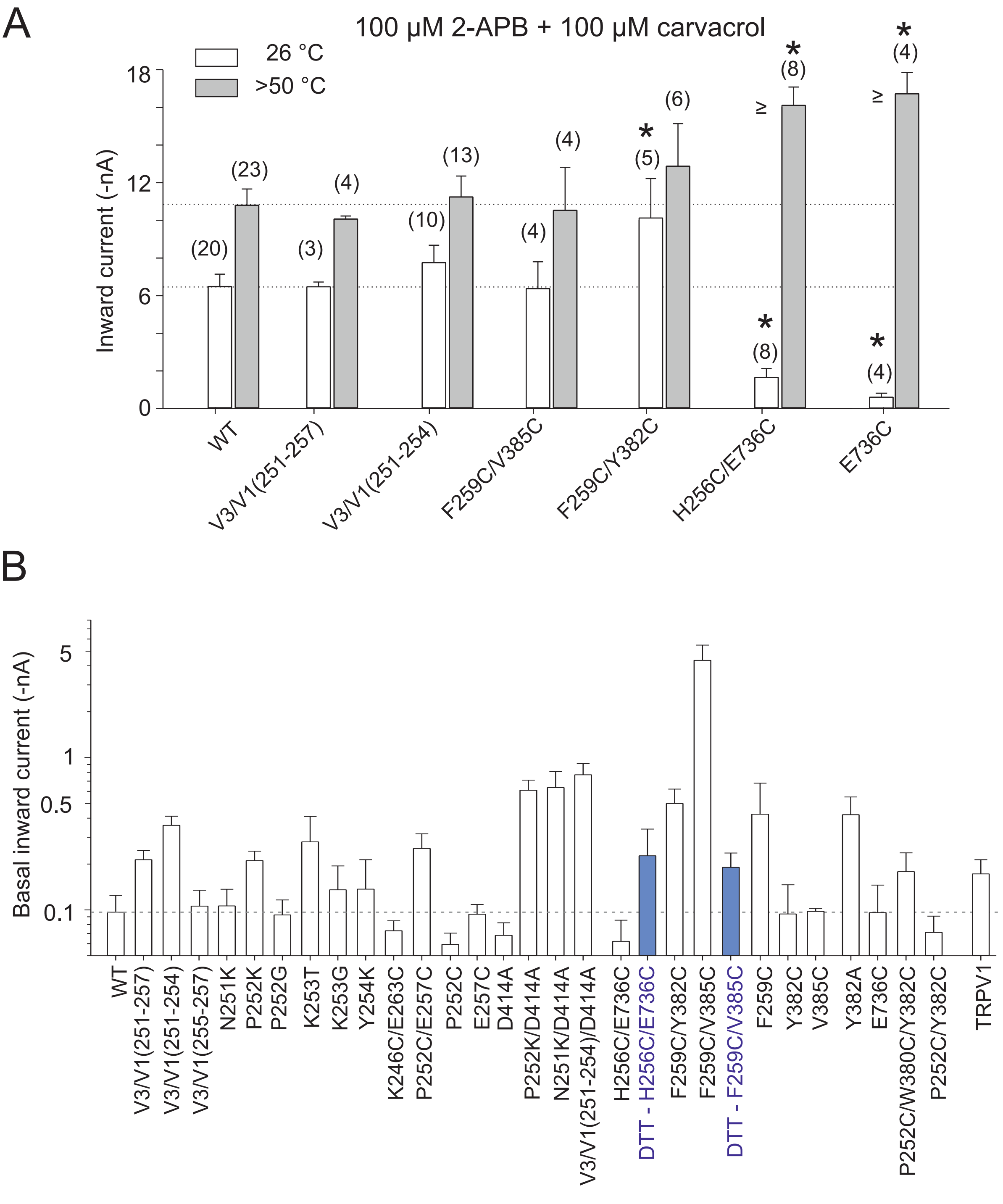

Figure S8. Basal inward current analysis reveals tonically active phenotypes. (A) Average instantaneous currents in response to a combination of agonists (100 µM 2-APB and 100 µM carvacrol) measured in wild-type (WT) and the indicated mutants at room temperature (25 °C) and above 50 °C. Number of cells is indicated in parentheses. (B) Comparison of average basal currents from all the constructs measured 30 s after whole cell formation at –70 mV. Number of cells 4-23. The basal current from HEK293T cells expressing wild type TRPV3 channels is indicated with a dotted horizontal line.

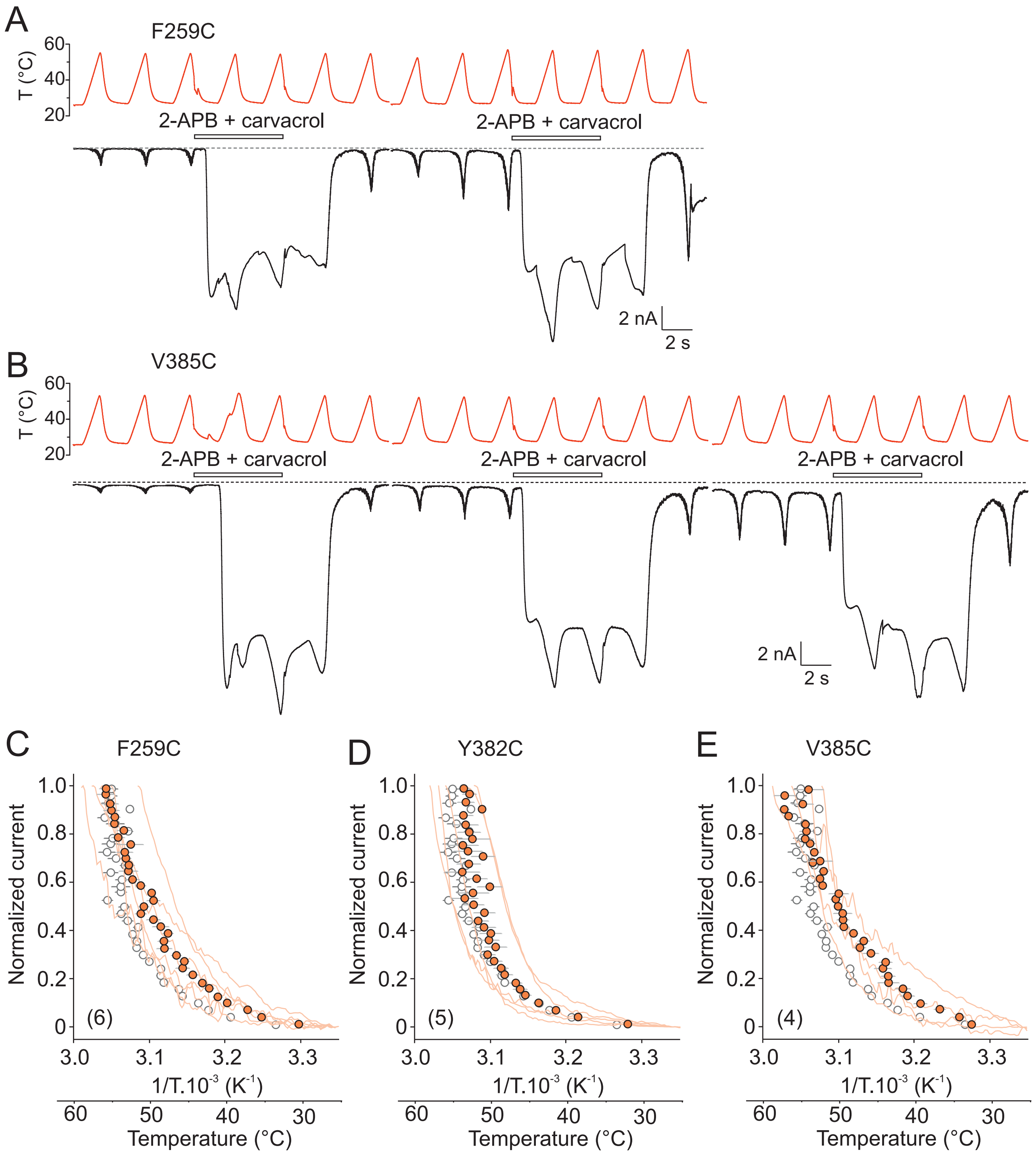

Figure S9. Control recordings to disulfide locking experiments. (A and B) Representative whole-cell currents evoked by repetitive heat stimuli (shown above the records) recorded from HEK293T cell expressing the indicated cysteine mutant channels. Currents were recorded in the absence or presence of mixed agonists (100 μM 2-APB + 100 μM carvacrol), as indicated by the horizontal bar above the records. Holding potential, -70 mV. (C-E) Average heat responses from HEK293T cells expressing the indicated mutants, normalized to the maximum amplitude with average values overlaid (colored circles with gray bi-directional error bars indicating S.E.M.). The average current for wild-type TRPV3 is overlaid for comparison as empty circles with grey bars indicating mean ± S.E.M. Number of cells is given in parentheses.

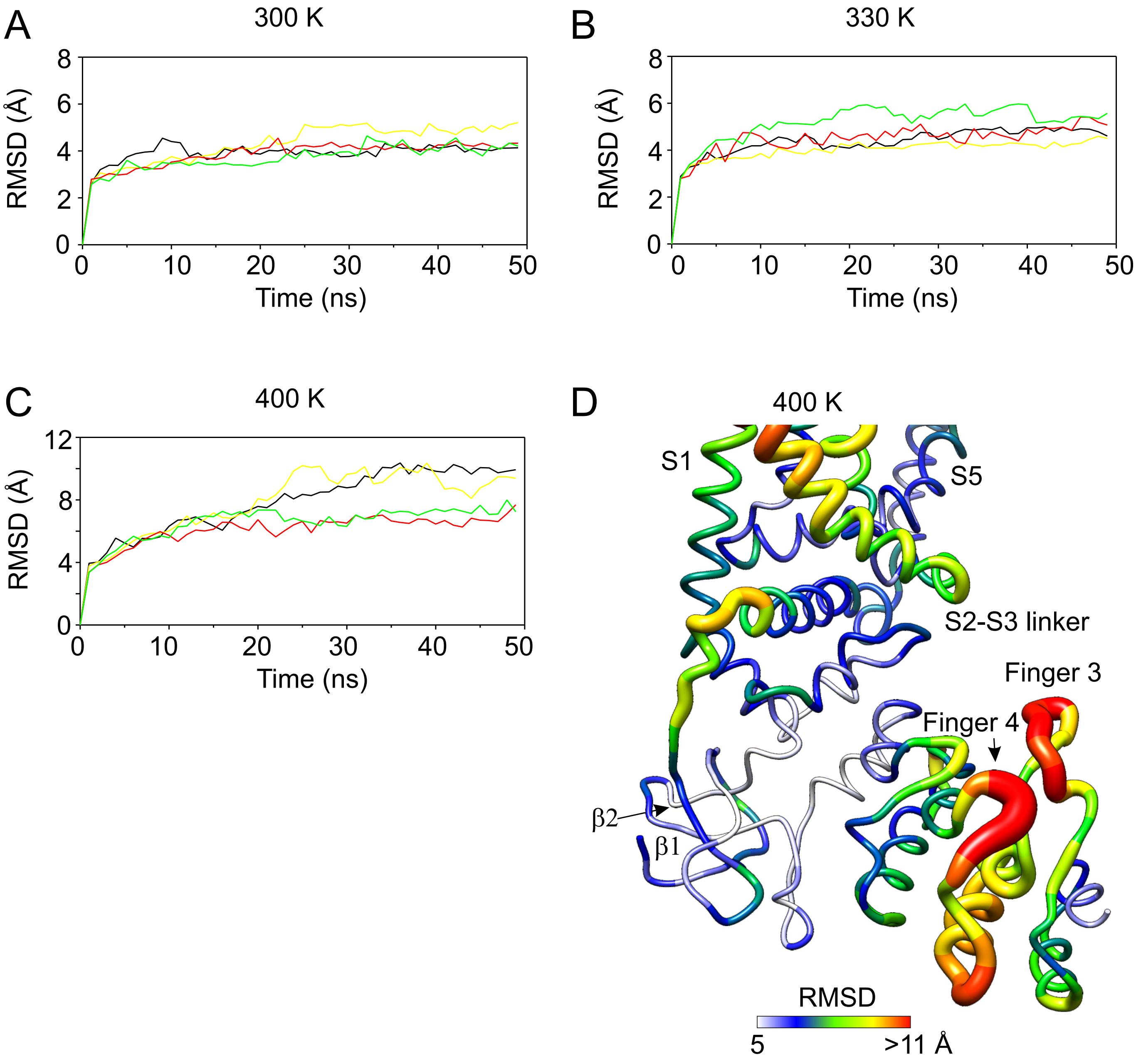

Figure S10. Time course of TRPV3 molecular dynamics simulations at three different temperatures. Closed-state TRPV3 structure (PDB ID: 6DVW) was subjected to 50-ns MD simulations at 300 K (~27 °C), 330 K (~57 °C) and 400 K (~127 °C). (A-C) RMSD for backbone atoms of four (colored differently) TRPV3 subunits at 300 K (A), 330 K (B) and 400 K (C). (D) The RMSD values calculated for the backbone atoms are shown for one monomer (chain A). The most flexible residues are colored red (RMSD >5 Å at 400 K), intermediately flexible yellow-to-green (>8 Å) and the least flexible blue-to-white (< 5 Å). The worm radius is proportional to RMSD (4.5 -13 Å).

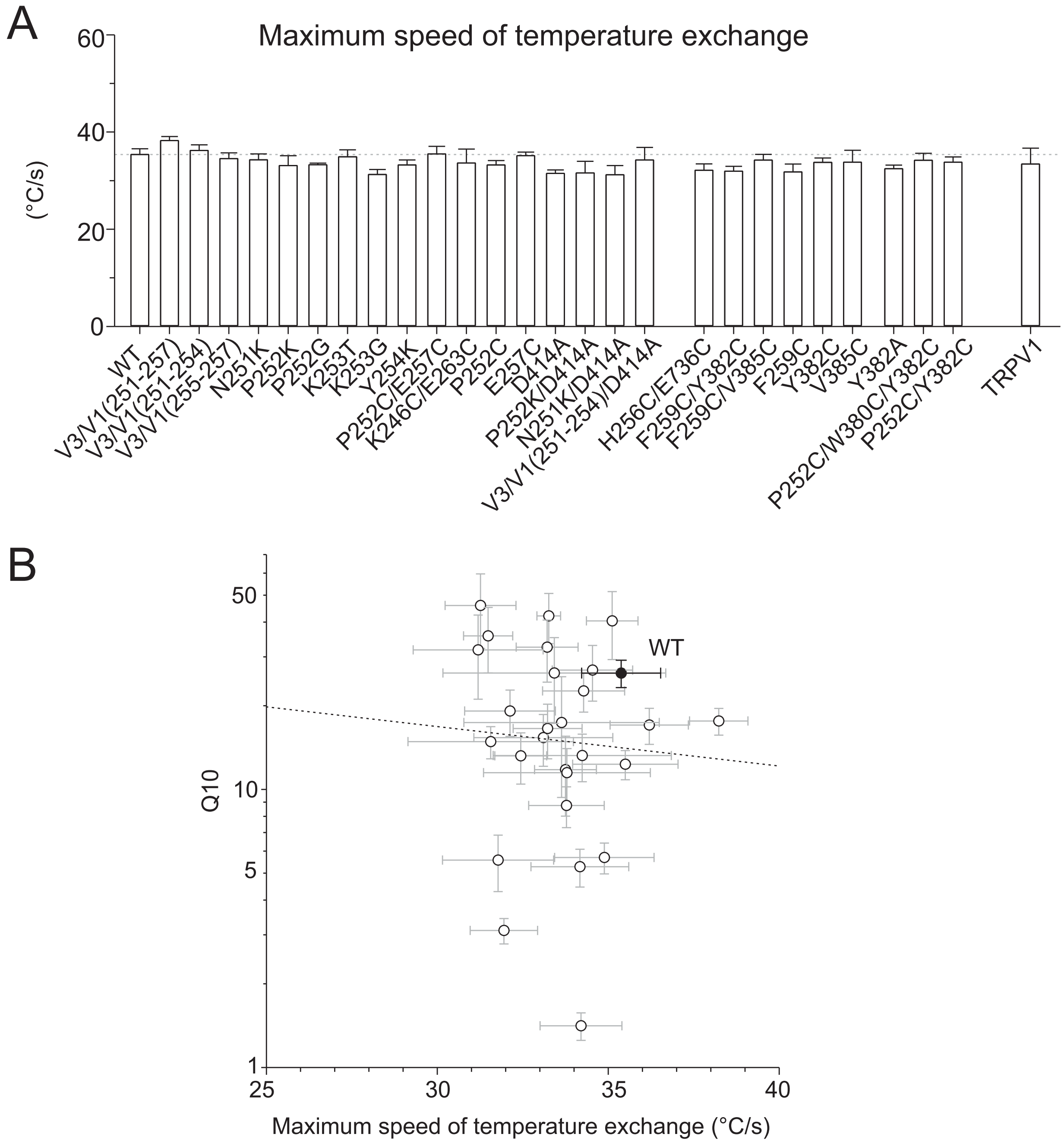

Figure S11. Comparison between the Q10 values and speed of temperature changes. (A) The average maximum speed of temperature changes measured in wild-type (WT) and mutant channels. No significant changes were detected. (B) The average speed of temperature changes did not correlate with the observed average Q10 value changes throughout all experiments. Pearson Product Moment and Spearman Rank Order correlation coefficients are -0.065 and -0.137 (P = 0.742 and 0.484, respectively; n = 28).
